# Low-Dose Radiation Therapy for Osteoarthritis: A Critical Review and Proposal for Integration Into Contemporary Care

**DOI:** 10.7759/cureus.116308

**Published:** 2026-09-15

**Authors:** Kishan M Patel, Trevor Wilson, Jessica Schuster, Bobby Koneru, Tarita O Thomas

**Affiliations:** 1 Radiation Oncology, Northwestern University Feinberg School of Medicine, Chicago, USA; 2 Radiation Medicine, University of Wisconsin–Madison, Madison, USA; 3 Radiation Oncology, University of Wisconsin–Madison, Madison, USA; 4 Radiation Oncology, Stritch School of Medicine, Cardinal Bernadin Cancer Center, Loyola University, Maywood, USA; 5 Radiation Oncology, UW Health in Northern Illinois, Rockford, USA

**Keywords:** knee osteo-arthritis, low-dose radiation therapy, multi-disciplinary teams, multimodal pain management, osteoarthritis (oa)

## Abstract

Osteoarthritis (OA) is a leading cause of pain and disability and continues to grow as a global health burden, yet many patients remain symptomatic despite guideline-directed pharmacologic, injectable, and surgical treatments. Low-dose radiation therapy (LDRT) has long been used in parts of Europe for benign painful joint disease and is now re-emerging as a noninvasive option for OA, although it remains underutilized and somewhat controversial in the United States. In this critical review, we synthesize mechanistic, clinical, and safety data for LDRT in OA, appraise standard therapies and key randomized controlled trials (RCTs) of LDRT, and position LDRT within a multidisciplinary care pathway. Preclinical and translational studies indicate that doses of 0.3-0.7 gray (Gy) per fraction (3-6 Gy total) reduce proinflammatory cytokine expression, blunt leukocyte-endothelial adhesion, and shift macrophages toward an anti-inflammatory phenotype, with concordant systemic immune changes documented in the immune modulation by low-dose radiation therapy (IMMO-LDRT01) cohort. Large European observational series report 60-80% pain response with minimal acute toxicity and a very low absolute rate of in-field second malignancy (three events, 0.064%, in a 4,699-patient orthovoltage cohort followed for a median of more than a decade) but are vulnerable to placebo effects and regression to the mean. Early sham-controlled RCTs were neutral, yet more recent randomized trials, including a double-blind sham-controlled study from Iran, the Korean low-dose radiation therapy for patients with knee osteoarthritis (LoRD-KNeA) trial, and a Russian RCT with a nine-year follow-up, demonstrate clinically meaningful improvements in pain and function and a possible reduction in arthroplasty and disability. Pooling the three sham-controlled trials that report an estimable between-group effect on pain yields a standardized mean difference of −0.19 (95% CI −0.72 to +0.33) with substantial heterogeneity (I² = 66%), indicating that the randomized signal is dose- and population-dependent rather than uniformly positive. We conclude that LDRT, prescribed as 3 Gy in six 0.5 Gy fractions, is a reasonable non-surgical option to offer selected patients aged ≥50 years with refractory OA as a second- or third-line option, provided outcomes are captured prospectively.

## Introduction and background

Osteoarthritis (OA) is a leading cause of pain and disability worldwide, with an estimated 595 million prevalent cases globally in 2020 and projected continued growth as populations age [1-3]. The most recent Global Burden of Disease analysis identifies OA as one of the largest and fastest-growing contributors to years lived with disability among non-communicable musculoskeletal disorders [3]. In the United States, approximately 1 in 5 adults reports a form of arthritis, and OA is the most common subtype [1-2]. Disease occurrence rises sharply with age and is more common among women and adults with obesity or metabolic disorders, with most cases occurring in adults aged 45 years and older [1-2]. Inadequately managed OA produces persistent pain, work and activity limitation, psychological distress, and substantial economic burden. In 2013, OA was the second most costly condition treated in U.S. inpatient settings and was associated with an estimated $164 billion in lost wages [4]. Demand for knee and hip OA care is projected to grow by 75-95% over the coming decades [1-2,5]. Despite its impact, no curative therapy exists, and management is directed at symptom control, function, and delay of structural progression.

OA is now understood as a complex whole-joint disease rather than purely mechanical wear-and-tear [6-7]. Progressive cartilage degradation, synovial inflammation, and dysregulated subchondral bone remodeling are accompanied by elevated proinflammatory cytokines, increased macrophage and leukocyte activity, suppressed osteoblast function, and increased osteoclast activity [6-7]. This evolving mechanistic picture has motivated interest in repurposing low-dose radiation therapy (LDRT). This is a modality with established anti-inflammatory and osteoimmunological effects in benign joint disease for symptomatic OA [6-9].

The contemporary OA armamentarium includes topical and oral nonsteroidal anti-inflammatory drugs (NSAIDs), intra-articular (IA) corticosteroid and hyaluronic acid (HA) injections, physical therapy, weight management, and ultimately joint arthroplasty for advanced disease [1,10-13]. Each option has limitations: NSAIDs carry cardiovascular, gastrointestinal, and renal risk and ~25% of patients do not achieve a meaningful response [10,14]; intra-articular corticosteroids provide only short-term relief and may accelerate cartilage loss with repeated use [14-16]; arthroplasty is highly effective but invasive, costly, and reserved for end-stage disease [10,17-18]. The 1976 U.S. approval of naproxen and the broader rise of NSAIDs likely contributed to the decline in U.S. use of LDRT for OA [19-20]. As the anti-inflammatory mechanism of LDRT has become better characterized, contemporary studies have shown clinically meaningful pain relief and functional improvement with little reported acute toxicity [19-20]. A retrospective German multicenter cohort of 970 patients with knee or hand OA demonstrated a 65.6% pain response at eight weeks [19], and a prospective hand-OA cohort reported approximately 94% improvement in pain and 70% improvement in function at six months [20].

LDRT remains underutilized in the United States because of unfamiliarity, concerns about secondary malignancy, and protocol heterogeneity [1,20]. The German Society for Radiation Oncology (DEGRO) recommends LDRT predominantly in patients over 40 years to minimize theoretical malignancy risk, with no convincing reports of radiation-induced cancer attributable to typical OA regimens [1,19,21-22]. Typical regimens deliver 0.3-0.7 Gy per fraction in six sessions, total 3-6 Gy [20-22]. This article compares the evidence for LDRT against that for other common OA interventions, topical and oral NSAIDs, intra-articular injections, genicular artery embolization (GAE), and arthroplasty, and proposes a multidisciplinary pathway in which LDRT is incorporated as a viable option for carefully selected patients [1,23].

## Review

Standard pharmacologic and injectable therapies

Acetaminophen

Acetaminophen (APAP) is often recommended as a first-line agent for mild-to-moderate OA pain because of its accessibility and favorable safety profile. It is thought to inhibit central cyclooxygenase (COX) pathways without meaningful peripheral anti-inflammatory effects. A 2019 Cochrane meta-analysis of 10 randomized trials (3,541 participants) found only a small reduction in pain compared with placebo, with a mean difference of -3.23 points on a 0-100 pain scale (95% confidence interval (CI) -5.43 to -1.02) at short-term follow-up (7 trials, 2,355 participants) and -3.32 (95% CI -5.83 to -0.81) in the immediate term (5 trials, 1,686 participants), both far below the review's prespecified 9-point minimal clinically important difference, and no measurable benefit on function or quality of life. All 10 included trials were prospective, double-blind, and placebo-controlled, comprising 7 parallel-group and 3 crossover designs. APAP was inferior to nonsteroidal anti-inflammatory drugs (NSAIDs) for OA pain [2,24]. Typical dosing is 4,000 mg/day in three to four divided doses [24]. While APAP carries a lower gastrointestinal and cardiovascular risk than NSAIDs, chronic or high-dose use is hepatotoxic, particularly in older adults or those with hepatic comorbidity [10]. Several contemporary guidelines have therefore deprioritized APAP for moderate-to-severe OA [1,12,25].

Intra-articular Corticosteroids

Intra-articular (IA) corticosteroid injections are commonly used to reduce IA inflammation and provide rapid pain relief and may be administered by orthopedists, rheumatologists, sports medicine physicians, or interventional pain specialists. A 2014 meta-analysis of 27 randomized controlled trials (RCTs) reported a significant reduction in pain at 1-2 weeks, with a standardized mean difference (SMD) of -0.41 (95% CI -0.61 to -0.21; p<0.001), an attenuated effect at 4-6 weeks (SMD -0.22; 95% CI -0.37 to -0.07; p=0.004), and no significant benefit at 13 weeks [15].

Contemporary trials and meta-analyses, including the 2017 randomized controlled trial by McAlindon and colleagues comparing IA triamcinolone with saline over two years in knee OA, have raised concern about both the durability and structural safety of repeated corticosteroid injections [16]. McAlindon et al. found that quarterly triamcinolone injections did not produce greater pain relief than saline at two years and were associated with significantly greater cartilage volume loss on magnetic resonance imaging (MRI) [16]. Additional longitudinal and imaging-based studies have linked repeated corticosteroid injections to accelerated cartilage loss, subchondral insufficiency fractures, and faster radiographic progression of OA, suggesting that short-term symptomatic gains may come at a structural cost [15-16]. Despite these signals, the 2019 American College of Rheumatology (ACR)/Arthritis Foundation guideline continues to conditionally recommend IA corticosteroids for short-term symptom relief in selected patients with knee OA, reflecting the lag between emerging structural-harm evidence and guideline updates [12]. Common adverse events include post-injection flare (2-10%), local infection (<0.1%), skin atrophy, and hypopigmentation; consensus guidance supports individualized, time-limited use, commonly avoiding administration more often than every 3 months per joint [12,15]. Taken together, the contemporary evidence supports a more cautious, time-limited, and individualized use of IA corticosteroids and motivates direct comparison with alternatives that may offer more durable benefit without accelerating structural damage.

Hyaluronic Acid (Viscosupplementation)

Hyaluronic acid (HA) injections aim to restore synovial fluid viscoelasticity, joint lubrication, and shock absorption. Evidence is mixed: a large 2022 systematic review and meta-analysis found that viscosupplementation was associated with only a small reduction in knee OA pain versus placebo, below the pre-specified threshold for a minimally clinically important between-group difference (24 trials, 8,997 participants; standardized mean difference -0.08, 95% CI -0.15 to -0.02, p=0.02, equivalent to approximately 2.0 mm on a 100 mm scale against a minimal important difference of -0.37), with ongoing debate about clinical significance [26]. Durability is a particular limitation: outcomes in that synthesis were assessed at a median of 13 weeks, and an earlier pooled analysis reported effect sizes of 0.43 (95% CI 0.26-0.60) at 4-13 weeks and 0.38 (95% CI 0.21-0.55) at 14-26 weeks, with essentially no controlled data extending beyond six months, so the frequently cited expectation of 6-12 months of benefit from a single HA course is not supported by randomized evidence [26-27]. Some data suggest HA may be more effective in younger patients with mild-to-moderate disease [27]. Adverse events are typically local pain, swelling, and rarely pseudoseptic reactions or infection, and serious adverse events were more frequent with viscosupplementation than placebo in the 2022 synthesis (3.7% vs 2.5%; relative risk 1.49, 95% CI 1.12-1.98) [26-27]. From a U.S. cost perspective, HA is associated with high acquisition costs and inconsistent payer coverage [26-27].

Nonsteroidal Anti-inflammatory Drugs

NSAIDs are widely used in OA management because of their analgesic and anti-inflammatory effects. Approximately 65% of OA patients are prescribed NSAIDs (about 55% nonspecific and 15% COX-2 inhibitors), and observational data suggest that more than 90% use NSAIDs at some point [28-29]. The 2019 Osteoarthritis Research Society International (OARSI) guideline recommends topical NSAIDs as a strong (level 1A) recommendation and oral NSAIDs as a conditional (level 1B) recommendation for first-line pharmacotherapy in OA [1]. NSAIDs block prostaglandin (PGE) production by inhibiting one or both COX isoforms; selective COX-2 inhibitors aim to limit gastrointestinal toxicity by sparing the gastroprotective COX-1 isoform [30].

A systematic review of 63 placebo-controlled RCTs reported that topical NSAIDs improved visual analog scale (VAS) pain by an average of 11.6 mm from a baseline of 54.7 mm on a 100 mm scale, with maximum effect at 1.6 weeks and decline thereafter [29]. Oral NSAIDs improved VAS pain by 10.2 mm from a baseline of 64.3 mm, with maximum effect at 2.3 weeks [29]. In contemporary U.S. prescribing data, meloxicam is the most frequently dispensed oral NSAID (approximately 19.7 million prescriptions annually), followed by ibuprofen (16.9 million), diclofenac (10.5 million), celecoxib (8.9 million), and naproxen (5.9 million), so the agents used most often in practice are not those with the largest measured analgesic effect [31]. Topical NSAIDs are typically more favorable than oral NSAIDs for pain control because they can be delivered at lower systemic doses with fewer adverse effects, and the minimum effective dose of topical diclofenac has a reported 92% probability of clinically relevant pain improvement [32]. Among oral NSAIDs, diclofenac 150 mg/day and etoricoxib 60 mg/day are most effective, with reported VAS reductions of 14 mm and 16 mm, respectively [32]. Efficacy is dose-dependent, with high-dose diclofenac outperforming maximum doses of ibuprofen, naproxen, and celecoxib [33]. Meta-analyses converge on the finding that oral NSAIDs deliver significant initial pain control over 1-4 weeks, with no measurable benefit beyond 4 weeks [29,34]; long-term data show no improvement at 1, 2, 3, or 4 years [35]. OARSI and ACR guidance recommend avoiding chronic oral or topical NSAID use [1,12,36].

Topical NSAIDs are associated mainly with minor skin reactions and have been reported to have no greater systemic adverse-event burden than placebo [1,34]. Oral NSAIDs carry well-established cardiovascular and gastrointestinal risks. A meta-analysis including more than 400,000 patients found that all NSAIDs increased myocardial-infarction risk in a dose-dependent manner, with reported odds ratios up to 1.58 after only 1-7 days of use [37]. This point warrants emphasis: the excess myocardial-infarction risk is measurable within the first week of exposure, before the analgesic effect reaches its maximum at 2-3 weeks, so there is no window of oral NSAID use that is simultaneously maximally effective and free of cardiovascular risk [29,37]. In a population with OA whose median age exceeds 65 years and in whom cardiovascular comorbidity is the rule rather than the exception, this is a first-order consideration rather than a footnote, and it is the single strongest argument for developing durable non-pharmacologic alternatives [1,37-38]. A network meta-analysis of 31 trials found increased stroke risk (odds ratio up to 3.36) and cardiovascular death (odds ratio up to 4.07) with NSAIDs [38]. Gastrointestinal complications, including significant bleeding, are increased across all NSAIDs, with odds ratios up to 3.97 versus placebo [39]. Chronic NSAID use, defined as >4 weeks, is also associated with renal impairment, particularly in older adults with pre-existing kidney disease or other risk factors [40]. OARSI guidance recommends avoiding oral NSAIDs in patients with cardiovascular comorbidity, and a COX-2 inhibitor is preferred for those with gastrointestinal risk [1,30].

While NSAIDs outperform placebo, the absolute pain reduction with topical and oral NSAIDs is small and only marginally above the minimal perceptible clinical improvement of 9.7 mm on a 100-mm VAS [29,34]. Fewer than 10% of patients are estimated to be able to distinguish an oral NSAID from placebo in selected analyses. Trials that do not exclude non-responders show reduced effect sizes (pain reduction of 0.23) [41], and most trials do not report data beyond four weeks, leaving long-term effectiveness in OA poorly characterized.

Arthroplasty

Total joint arthroplasty produces large gains in pain and function for end-stage OA but is invasive and is associated with perioperative morbidity, revision risk, and prolonged recovery [10,17-18]. In current U.S. Medicare pricing, primary total knee arthroplasty is reimbursed at approximately $10,552 in the ambulatory surgical center setting and $14,275 in the hospital outpatient setting, compared with approximately $77-$352 for an intra-articular injection and $5,901-$12,276 for genicular artery embolization [10,17-18,42]. Importantly, LDRT and arthroplasty are not mutually exclusive and do not compete for the same point in the pathway. LDRT delivers 3-6 Gy to a small orthovoltage or low-energy photon field, does not violate the joint capsule, does not alter bone stock or soft-tissue planes, and has not been reported to compromise subsequent arthroplasty, wound healing, or implant survival [21-22,43]. LDRT should therefore be positioned as a means of deferring or avoiding surgery in patients who are not yet surgical candidates, not as a treatment that forecloses later surgery; patients who progress despite LDRT remain fully eligible for arthroplasty. A retrospective series presented at the American College of Radiation Oncology has additionally explored LDRT in the distinct postoperative setting of aseptic synovitis after total knee arthroplasty, using 6-10 MV field-in-field planning with heterogeneity corrections and WOMAC assessment at baseline, at the end of radiotherapy, and at approximately 90 days, suggesting a possible role for LDRT after as well as before arthroplasty [44]. Many patients are poor surgical candidates because of comorbidity or prefer to defer surgery, highlighting the need for durable intermediate options [5].

Genicular Artery Embolization

Genicular artery embolization (GAE) is considered here after arthroplasty because, unlike joint replacement, it is not an established therapy but an investigational procedure whose role is defined largely in relation to surgery. It is an emerging catheter-based, minimally invasive therapy for knee OA that targets synovial hypervascularity through selective embolization of geniculate branches supplying inflamed synovium. Prospective single-arm series have reported clinically meaningful reductions in pain and improvements in function, but the best available sham-controlled randomized trial did not establish a clinical effect, with similar Knee Injury and Osteoarthritis Outcome Score (KOOS) pain improvements after GAE and sham at 4 months (between-group difference 3.0 points, 95% CI -7.1 to 13.0; Cohen d 0.15; p=0.31) and no serious adverse events [45]. The between-group difference remained non-significant at 12 months (7.46 points, 95% CI -13.63 to 28.56; p=0.25), and adverse events were more frequent after GAE than after sham (23 vs 5 events), most commonly transient skin erythema (11 patients) and lateral-ankle paraesthesia (2 patients, persistent in 6.9%) [45-46]. A 2025 systematic review of sham-controlled RCTs concluded that GAE appears safe and may provide short-term pain reduction in selected trials, but emphasized mixed functional outcomes, heterogeneity in technique and trial design, and only three sham-controlled RCTs totaling 138 patients [46]. Procedurally, GAE requires arterial access, fluoroscopic guidance, and an embolic agent; its combined technical and professional cost in the United States is broadly comparable to a course of LDRT, but the risk profile is procedural rather than radiologic [42,46]. Major rheumatology and orthopedic guidelines have not yet incorporated GAE as routine therapy, and its use remains concentrated in specialized interventional radiology centers and clinical trials. For the purposes of this review, GAE is best viewed as an investigational or niche interventional option that may "bridge the gap" between injections and arthroplasty for selected patients who are poor surgical candidates or strongly averse to joint replacement. The comparative role relative to LDRT will depend on additional multicenter sham-controlled data [45-46].

Comparative Summary of Standard Therapies

When compared with LDRT, standard OA therapies show meaningful gaps in durability and safety that LDRT may help fill. The German multicenter cohort of 970 elderly patients with knee or hand OA reported a 65.6% pain response at 8 weeks following LDRT [19], and a prospective hand-OA cohort observed 94% pain improvement and 70% functional improvement at 6 months [20]. Compared with corticosteroid and HA injections, these data suggest longer-lasting pain relief, minimal acute toxicity, and no reported radiation-attributable malignancies in older adults [19-22]. The upfront cost of LDRT in the United States is non-trivial: institutional estimates for a six-fraction course cluster in the range of approximately $4,000-$8,000 in combined professional and technical charges, more than a course of injections but substantially less than arthroplasty [19-20,42]. We are aware of no published formal cost-effectiveness or cost-utility model of LDRT in OA. The proposition that a longer duration of effect and a reduced need for repeated interventions would translate into cost-effectiveness therefore remains an untested hypothesis rather than a demonstrated finding, and formal economic modeling incorporating quality-adjusted life years and avoided or deferred arthroplasty is a specific and currently unmet research need [19-20,23,42]. Figure 1 presents a comparative analysis of the duration and magnitude of symptom relief across OA therapies, and Table 1 summarizes the comparative profile of the major OA interventions.

**Figure 1:**
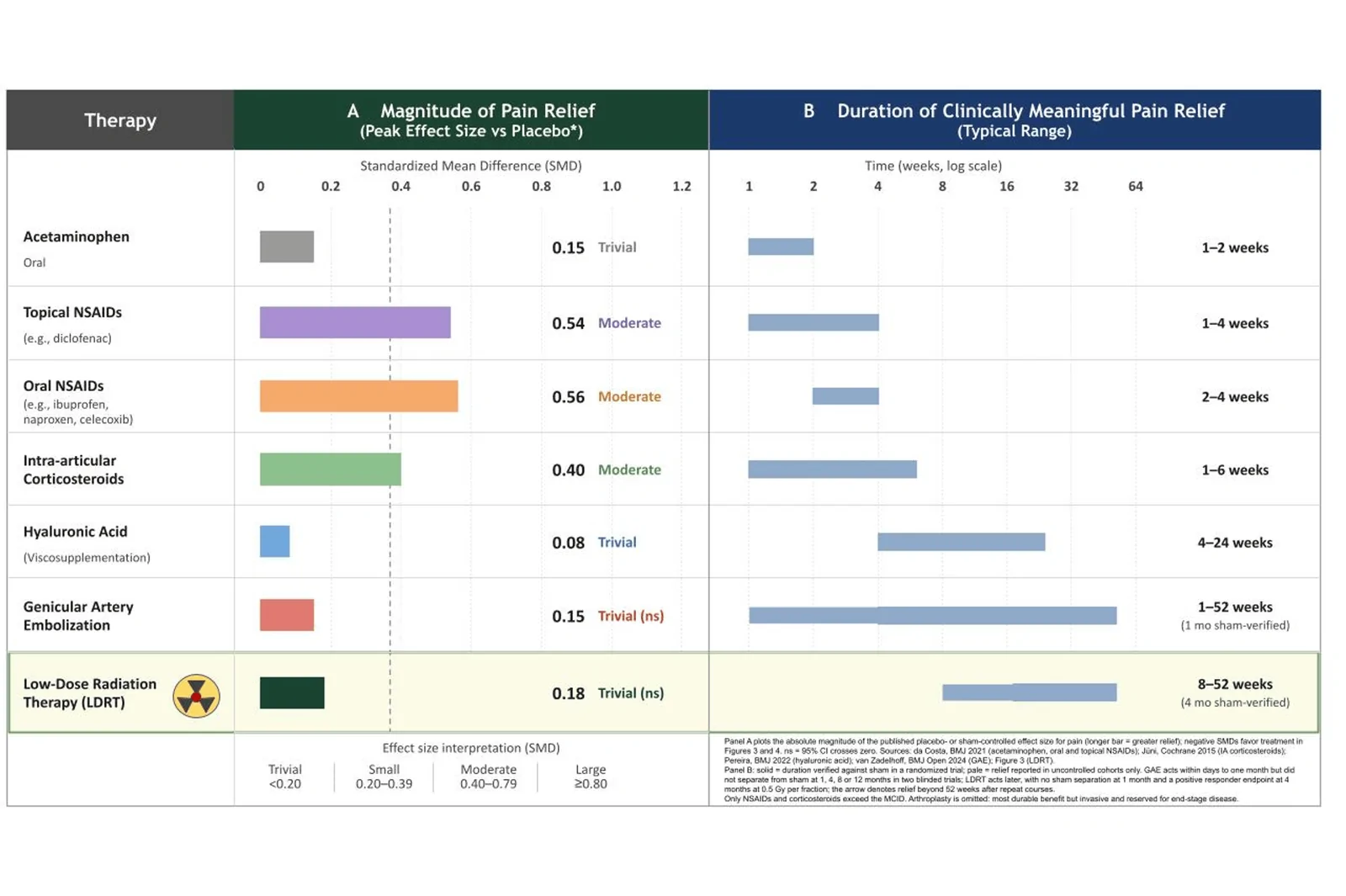
Comparative duration and magnitude of symptom relief across common osteoarthritis therapies Panel A shows the magnitude of pain relief for seven osteoarthritis therapies as the published placebo- or sham-controlled standardized mean difference, plotted as absolute magnitude, so a longer bar denotes greater relief. For the underlying analyses, negative values favor treatment; the analyses are presented later in the article. Categories follow conventional thresholds (trivial <0.20, small 0.20-0.39, moderate 0.40-0.79, large ≥0.80). Plotted values and sources: acetaminophen 0.15, topical NSAIDs 0.54, oral NSAIDs 0.56 (da Costa, BMJ 2021); IA corticosteroids 0.40 (Jüni, Cochrane 2015); hyaluronic acid 0.08 (Pereira, BMJ 2022); GAE 0.15 (van Zadelhoff, BMJ Open 2024); LDRT 0.18 (please refer to the forest plot of the pooled sham-controlled estimate). The suffix ns marks estimates whose 95% confidence interval crosses zero. The dashed line marks the minimal clinically important difference of 0.37 SD (approximately 9 mm/100 mm); only NSAIDs and corticosteroids exceed it. Panel B shows the typical duration of clinically meaningful benefit on a logarithmic time axis in weeks; solid segments denote durability verified against sham, and pale segments denote relief from uncontrolled cohorts only. GAE acts within days to one month (1-52 weeks plotted) but did not separate from sham at 1, 4, 8, or 12 months, whereas LDRT acts later (8-52 weeks plotted), with no separation at 1 month and a positive sham-controlled responder endpoint at 4 months at 0.5 Gy per fraction; the arrow denotes benefit reported beyond 52 weeks after repeat courses. Arthroplasty is not plotted: its benefit is the largest and most durable, but it is invasive and reserved for end-stage disease. Abbreviations: GAE = genicular artery embolization; IA = intra-articular; LDRT = low-dose radiation therapy; NSAIDs = nonsteroidal anti-inflammatory drugs. Image credit: original figure created by the authors using Microsoft PowerPoint and Google Slides.

**Table 1:** Comparative summary of osteoarthritis interventions. Duration reflects the typical range reported in the literature; for genicular artery embolization and LDRT the interval verified against sham is given in parentheses, with longer intervals derived from uncontrolled cohorts. COX, cyclooxygenase; CV, cardiovascular; GI, gastrointestinal; IA, intra-articular; LDRT, low-dose radiation therapy; RCT, randomized controlled trial. References: [10,15-20,23-24,26,28-29,37-39,45-46]

Intervention	Mechanism	Peak effect	Duration	Key limitations
Acetaminophen	Central COX inhibition	1-2 weeks	1-2 weeks	Effect below MCID (MD -3.3/100); hepatotoxicity
IA corticosteroids	Local anti-inflammatory	1-2 weeks	1-6 weeks	Short duration; possible cartilage harm
Hyaluronic acid	Viscosupplementation	4-12 weeks	4-24 weeks	Pooled effect below MCID; high cost
Topical NSAIDs	Local COX inhibition	1-2 weeks	1-4 weeks	Modest benefit; skin reactions
Oral NSAIDs	Systemic COX inhibition	2-3 weeks	2-4 weeks	CV/GI/renal risks; short duration
Arthroplasty	Joint replacement	Immediate	Years	Invasive; perioperative risk; recovery
Genicular artery embolization	Synovial devascularization	1 day-1 month	1-12 months (only 1 month sham-verified)	Sham-controlled KOOS difference not significant at 1, 4, 8 or 12 months; specialized
LDRT	Immune modulation; osteoimmunology	6-12 weeks	2-12 months (4 months sham-verified)	Benefit sham-verified only at 0.5 Gy/fraction; 1.0 Gy/fraction trials neutral; late malignancy risk unquantified

Pathophysiology of osteoarthritis

OA is increasingly recognized as a complex whole-joint disease rather than purely "wear-and-tear" [6-7,47]. Synovial inflammation, innate immune activation, and dysregulated bone remodeling proceed alongside cartilage degeneration. Synovial fibroblasts, macrophages, chondrocytes, osteoclasts, and osteoblasts interact through cytokines and chemokines, including tumor necrosis factor alpha (TNF-α), interleukin (IL)-1β, and IL-6, to drive pain, stiffness, osteophyte formation, and progressive structural damage [6-7]. Proinflammatory macrophage polarization, increased leukocyte adhesion, and osteoclast-mediated bone resorption are prominent features of symptomatic disease and plausible targets for LDRT.

Radiobiological rationale for LDRT in OA

LDRT is a non-pharmacologic modality with biologic effects that differ qualitatively from oncologic high-dose radiation [6,9,30,48]. Preclinical studies show that doses of 0.3-1.0 Gy per fraction (total 3-6 Gy) exert anti-inflammatory and osteoimmunological effects rather than directly cytotoxic effects [6,48-49]. The dose-response relationship for these anti-inflammatory endpoints is discontinuous rather than monotonic, with maximal effects clustering near 0.5 Gy per fraction and attenuation at both lower and higher single doses; this non-linearity has been characterized for leukocyte-endothelial adhesion, nitric oxide and transforming growth factor beta signaling, and apoptosis induction within the 0.3-0.7 Gy window [50-51]. It should be distinguished from low-dose hyper-radiosensitivity and induced radioresistance, which are clonogenic cell-survival phenomena described between approximately 0.1 and 0.6 Gy in tumor and normal cell lines and which are not the mechanistic basis for the anti-inflammatory optimum; the reduced efficacy of 1 Gy relative to 0.5 Gy per fraction observed in the randomized data is better explained by the discontinuous anti-inflammatory dose-response than by hyper-radiosensitivity [50-52]. Experimental models demonstrate that low-dose ionizing radiation reduces expression of adhesion molecules and proinflammatory cytokines (TNF-α, IL-1β, IL-6), diminishes leukocyte-endothelial interactions, and shifts macrophages from a proinflammatory (M1-like) toward an anti-inflammatory (M2-like) phenotype [6,30,48-49]. In parallel, LDRT decreases osteoclast number and activity while preserving or enhancing osteoblast function, suggesting a bone-protective effect relevant to osteophyte formation and subchondral remodeling in OA [6,49,53].

In the IMMO-LDRT01 translational study, 125 patients of a planned 250 with degenerative and inflammatory musculoskeletal conditions were treated with orthovoltage LDRT to a total dose of 3 Gy in six 0.5 Gy fractions and serially immunophenotyped [8]. The cohort had a mean age of 58 years (range 41-83) and was 74.4% female; the most common single indication was calcaneodynia (45 patients), and only 16 patients (12.8%) were treated for osteoarthritis, so the immunological findings derive predominantly from non-OA degenerative indications and should be extrapolated to OA with caution [8]. Pain on a visual analog scale fell from a mean of 6.5 to 3.8 [8]. As reported in the published corrigendum to that analysis, circulating eosinophils, basophils, and plasmacytoid dendritic cells increased while B cells decreased, monocyte CD25 and HLA-DR expression decreased, and programmed cell death protein 1 expression on T-helper cells decreased; no cytokine measurements were reported, so the claim of reduced circulating proinflammatory cytokines in this specific cohort is not supported by the primary publication and rests instead on the preclinical literature cited above [8,54]. These data support a model in which LDRT remodels joint and periarticular inflammatory networks rather than simply providing transient nociceptive blockade. Figure 2 illustrates the proposed mechanisms of LDRT in OA.

**Figure 2:**
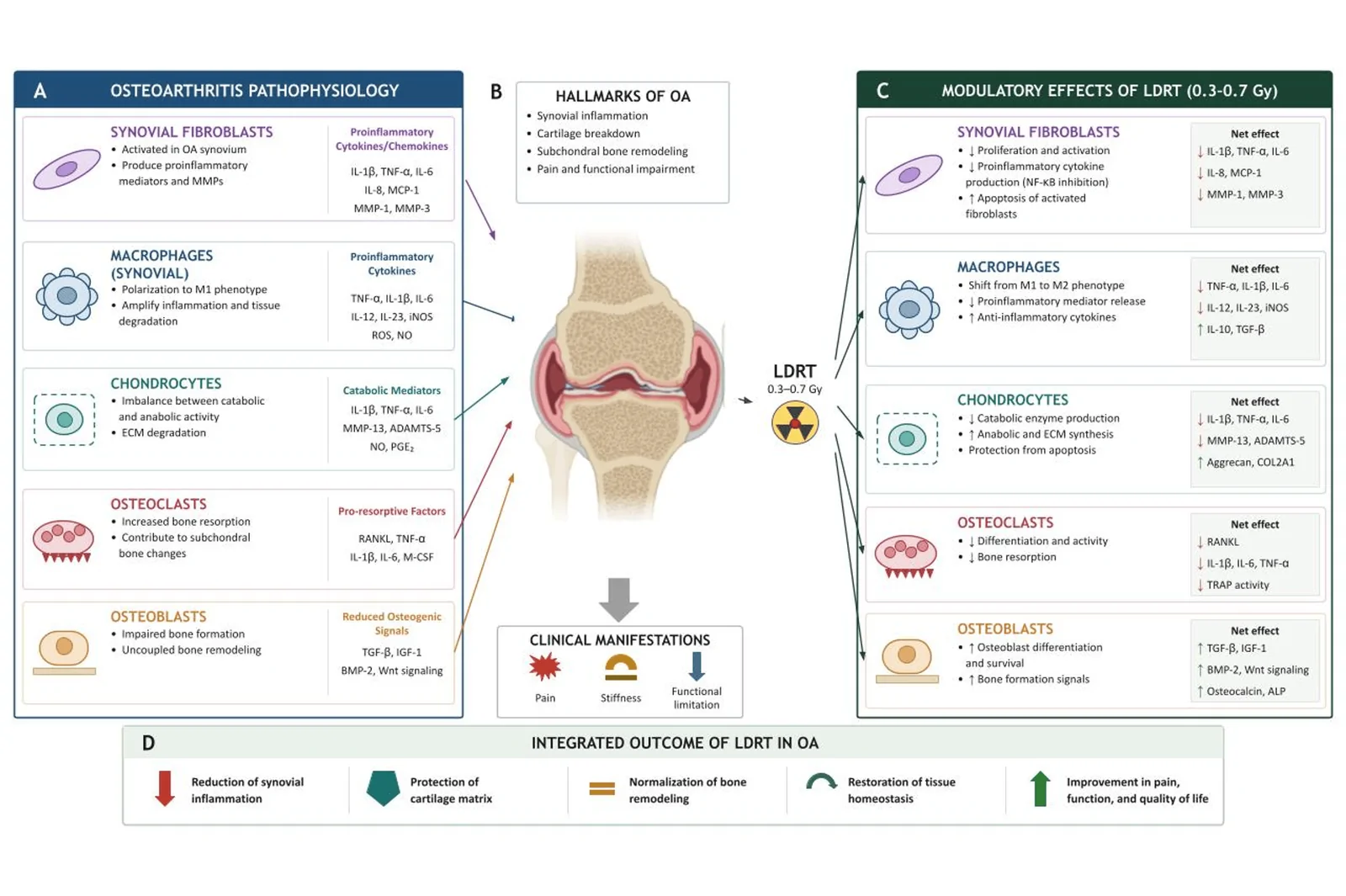
Pathophysiology of osteoarthritis and proposed mechanisms of action of low-dose radiation therapy. Panel A summarizes the cellular drivers of osteoarthritis: for each of five cell types (synovial fibroblasts, synovial macrophages, chondrocytes, osteoclasts, and osteoblasts), it lists the disease-associated behavior and the principal mediators released or lost. Panel B shows the resulting joint-level hallmarks, the affected knee joint, the fractional dose range associated with maximal anti-inflammatory effect in preclinical studies (0.3-0.7 Gy per fraction; the sham-controlled trials used 0.5-1.0 Gy), and the clinical manifestations of pain, stiffness, and functional limitation. Panel C shows the proposed modulatory effects of low-dose radiation therapy on the same five cell types, with the net direction of change in each mediator indicated by downward (decreased) or upward (increased) arrows. Panel D summarizes the integrated therapeutic outcome. All effects shown are mechanistic and are derived from the preclinical and translational studies discussed in the main text. Abbreviations: ADAMTS-5 = a disintegrin and metalloproteinase with thrombospondin motifs 5; ALP = alkaline phosphatase; BMP = bone morphogenetic protein; COL2A1 = collagen type II alpha 1 chain; ECM = extracellular matrix; Gy = gray; IGF-1 = insulin-like growth factor 1; IL = interleukin; iNOS = inducible nitric oxide synthase; LDRT = low-dose radiation therapy; M-CSF = macrophage colony-stimulating factor; MCP-1 = monocyte chemoattractant protein 1; MMP = matrix metalloproteinase; NF-κB = nuclear factor kappa B; NO = nitric oxide; OA = osteoarthritis; PGE₂ = prostaglandin E₂; RANKL = receptor activator of nuclear factor κB ligand; ROS = reactive oxygen species; TGF-β = transforming growth factor beta; TNF-α = tumor necrosis factor alpha; TRAP = tartrate-resistant acid phosphatase. Image credit: Original figure created by the authors using Microsoft PowerPoint, Google Slides, and BioRender.

Clinical evidence for LDRT in OA

Observational Cohorts and Contemporary Series

Modern retrospective and prospective cohorts from Europe and elsewhere have evaluated LDRT for symptomatic OA, typically using total doses of 3-6 Gy delivered as 0.5-1.0 Gy fractions over six sessions [19-20,43]. The previously described German multicenter analysis of 970 elderly patients (1,185 joints) reported a 65.6% pain response at the first follow-up assessment approximately 8 weeks after treatment, with a 60.0% response already recorded immediately at the end of the radiotherapy course; that series did not include a three-month assessment, and earlier descriptions of a three-month response rate in this cohort were in error [19]. The median patient age was 76 years (range 65-98), 77.3% of treated sites received 6 × 1 Gy and 21.7% received 6 × 0.5 Gy, and 384 sites (32.4%) were re-irradiated [19]. Acute toxicity was neither graded nor tabulated in that series, which reported only a near absence of toxicities, so it cannot be used to quantify toxicity rates [19]. A separate multi-joint analysis reported significant and durable pain reduction across various OA locations, with many patients maintaining benefit at 12 months [43]. Prospective data reinforce these findings: a hand-OA cohort treated with LDRT reported approximately 94% pain improvement and 70% functional improvement at six months, with negligible acute toxicity [20]. An American single-institution retrospective series using 0.5 Gy × 6 fractions across multiple joints similarly demonstrated clinically meaningful and sustained pain reductions [55]. Although these studies lack sham controls and are subject to regression to the mean and placebo effects, the consistency, magnitude, and durability of improvement across centers and joint sites are notable [19-20,43,55].

Randomized and Sham-Controlled Trials

Two early sham-controlled RCTs, together with their pooled long-term follow-up, have shaped skepticism around LDRT in OA. Minten et al. randomized 56 patients with painful hand OA to 6 × 1 Gy (total 6 Gy) versus sham irradiation and found that both arms improved at three months without a significant between-group difference; an OMERACT-OARSI response was achieved in 8 of 28 patients (29%) after LDRT versus 10 of 28 (36%) after sham (difference -7%, 95% CI -31% to +17%), and the between-group estimate for Australian/Canadian Hand Osteoarthritis Index pain was +4.5 (95% CI -3.4 to 12), numerically favoring sham [56]. The authors explicitly advised against the use of LDRT for hand OA on the basis of these results [56]. Mahler et al. used an identical 6 × 1 Gy regimen in knee OA and likewise observed no clinically meaningful difference between LDRT and sham at three months despite improvement in both groups; response rates were 12 of 27 (44%) versus 12 of 28 (43%), p=0.9, and the between-group estimate for WOMAC pain was -3 (95% CI -10 to +4) [57]. Extended follow-up reported by van den Ende et al. pooled these same two randomized cohorts at 6 and 12 months rather than constituting an independent third trial, and showed sustained symptom improvement in both arms with no late separation favoring LDRT (knee response 13 of 25, 52%, versus 11 of 25, 44%, odds ratio 1.41, 95% CI 0.45-4.48; hand response 8 of 26, 31%, versus 7 of 26, 27%, odds ratio 1.23, 95% CI 0.37-4.12); the authors attributed the within-group gains seen in both arms to placebo response and regression to the mean [58]. Because these are the same patients, this report must not be counted as a separate study in any pooled analysis, and it is excluded from the meta-analysis presented in Figure 3.

More recent trials have addressed several limitations of these earlier studies. The ArthroRad randomized multicenter single-blinded trial compared two active LDRT regimens in painful OA (without a sham arm) and reported favorable pain relief over 12 months, confirming that contemporary regimens are well tolerated and associated with sustained benefit [59]. Fazilat-Panah et al. performed a double-blind, sham-controlled RCT in older adults with radiographically confirmed knee OA using a total dose of 3 Gy delivered in six 0.5 Gy fractions in addition to usual care. In contrast to the earlier trials, the LDRT arm showed significantly greater improvement in pain, function, and analgesic use from one through six months, with no adverse effects reported [60]. This trial reports within-group change over time rather than an adjusted between-group treatment effect with a confidence interval, so it cannot contribute a standardized mean difference to a between-group meta-analysis and is excluded from the pooled estimate in Figure 3 [60].

The Korean low-dose radiation therapy for patients with KOA (LoRD-KNeA) multicenter randomized sham-controlled trial enrolled 114 patients with mild-to-moderate knee OA (Kellgren-Lawrence grade 2-3) across three academic centers. Patients were randomized 1:1:1 to sham, 0.05 Gy × 6 fractions (total 0.3 Gy), or 0.5 Gy × 6 fractions (total 3 Gy), with analgesic use restricted to acetaminophen during follow-up to maximize attribution of outcomes to treatment [61]. Eligibility required walking pain of 50-90 on a 100 mm scale [61]. At 4 months, the 3 Gy total-dose arm demonstrated a significantly higher Outcome Measures in Rheumatology combined with the Osteoarthritis Research Society International (OMERACT-OARSI) responder rate than sham (70.3%, 26 of 37, versus 41.7%, 15 of 36; p=0.014; odds ratio 3.31, 95% CI 1.26-8.71) and a greater proportion achieving the minimal clinically important difference in Western Ontario and McMaster Universities Osteoarthritis Index (WOMAC) total score (56.8% vs 30.6%; p=0.024), with no treatment-related toxicity in any arm [61]. The trial did not, however, demonstrate a significant between-arm difference in mean visual analog scale pain, patient global assessment, or circulating inflammatory markers, so the positive result rests on dichotomous responder endpoints rather than on mean symptom scores [61]. Notably, the 0.3 Gy per-fraction arm did not separate from sham (58.3% versus 41.7%; p=0.157), providing dose-differentiation evidence supporting 3 Gy total (0.5 Gy × 6) over very low-dose regimens [61]. Twelve-month follow-up data, including durability and imaging endpoints, have not yet been reported. In parallel, the prospectively registered Knee Osteoarthrosis Radiation Trial (KORT) protocol describes an ongoing pragmatic randomized investigation of LDRT for knee OA, which will further inform optimal dose, patient selection, and long-term outcomes [62].

A randomized controlled trial by Makarova and colleagues provides key long-term data on surgical endpoints in early-stage knee OA. In 292 patients with Altman-defined symptomatic knee OA and Kellgren-Lawrence 0-2, standard therapy with NSAIDs plus symptomatic slow-acting drugs for OA (SYSADOA) was compared with the same regimen plus LDRT to a total dose of 4.5 Gy delivered as 10 fractions of 0.45 Gy at 48-hour intervals [23]. Over 9 years of follow-up (2,131.2 person-years), knee arthroplasty was performed in 4.1% (6/146) of LDRT patients versus 7.5% (11/146) of controls, an incidence density ratio of 0.60 (95% CI 0.18-1.88) which is approximately a 40% relative risk reduction that did not reach statistical significance because of the small number of events (p=0.34) [23]. Neither the primary trial report nor the companion disability analysis reports a prospective sample-size or power calculation for the arthroplasty endpoint, and with only 17 arthroplasty events across both arms, the trial was neither designed nor adequately powered to detect a difference in arthroplasty incidence [23,63]. We deliberately refrain from presenting a post-hoc power calculation, because power computed retrospectively from the observed effect is uninformative and can be misleading; we note instead that detecting the observed incidence density ratio of 0.60 with 80% power at a two-sided alpha of 0.05 would require on the order of several hundred events, and therefore several-fold more patients or person-years than the 2,131.2 person-years accrued here. The arthroplasty and disability findings should accordingly be read as exploratory signals that justify a prospectively powered replication trial, not as evidence that LDRT reduces the need for arthroplasty. Attributable and population-attributable fraction analyses suggested, if confirmed, that LDRT could prevent about 40% of arthroplasties in similar trial populations and about 21% in the broader population of patients with radiographic stage 0-2 knee OA [23]. A companion analysis from the same cohort evaluated long-term disability: 9.5% of LDRT recipients became disabled over a mean 10-year follow-up versus 17.8% in the standard-care arm (incidence rate ratio 0.50; 95% CI 0.25-0.99; p=0.018), and after adjustment for age, sex, baseline radiographic grade, pain intensity, and OA duration, the hazard ratio for disability was 0.24 (95% CI 0.11-0.48), implying roughly a 75% relative reduction [63]. Population-level modeling suggested disability could be reduced by about 50% in similar trial populations and by about 25% in the broader population if orthovoltage radiotherapy were widely implemented [63]. These analyses are best interpreted as hypothesis-generating but are notable as the first randomized evidence suggesting that adjunct LDRT may delay or reduce arthroplasty and disability in early-stage disease.

Regarding the derivation of the standardized mean differences shown in Figure 3, because the primary trials report adjusted between-group mean differences on instrument-specific scales rather than standardized effect sizes, each standardized mean difference was obtained by dividing the published adjusted between-group mean difference by the pooled baseline standard deviation of that trial's pain instrument, and the 95% confidence limits were divided by the same value. For Minten et al., the AUSCAN pain difference of +4.5 (95% CI -3.4 to 12) at three months was divided by a pooled baseline standard deviation of 17.12 (LDRT mean 54, SD 19; sham mean 56, SD 15), giving +0.26 (95% CI -0.20 to +0.70) [56]. For Mahler et al., the WOMAC pain difference of -3 (95% CI -10 to +4) at three months was divided by a pooled baseline standard deviation of 15.60 (LDRT mean 59, SD 14; sham mean 61, SD 17), giving -0.19 (95% CI -0.64 to +0.26) [57]. For the 3 Gy arm of the LoRD-KNeA trial, the four-month OMERACT-OARSI responder counts of 26 of 37 versus 15 of 36 (odds ratio 3.31, 95% CI 1.26-8.71) were converted to a standardized effect size using the Chinn transformation, d = ln(odds ratio) × √3/π, giving -0.66 (95% CI -1.19 to -0.13) [61]. Fazilat-Panah et al. report only within-group change over time and no adjusted between-group estimate and are therefore not estimable [60]. The van den Ende extended follow-up reports the same Minten and Mahler cohorts at 6 and 12 months and is excluded from pooling to avoid double-counting patients [58]. Pooling used inverse-variance weighting, with between-study variance for the random-effects model estimated by the DerSimonian-Laird method; each of the three estimable trials contributed approximately one third of the weight. For the comparison with total knee arthroplasty shown in Figure 4, no pooled standardized mean difference has been published; we therefore derived one from the randomized trial of Skou et al., in which total knee arthroplasty followed by 12 weeks of non-surgical treatment improved the mean KOOS4 score by an adjusted 15.8 points more than non-surgical treatment alone (95% CI 10.0 to 21.5) in 100 patients randomized 1:1 [64]. Converting that confidence interval to a standard error of 2.93 and, with 50 patients per arm, to a pooled standard deviation of 14.67 gives a standardized mean difference of -1.08 (95% CI -1.47 to -0.68), which the Hedges g small-sample correction changes only to -1.07. This value is derived by the authors rather than published and is labeled as such in Figure 4.

Figure 3 presents a forest plot of the randomized sham-controlled trials of LDRT in OA that report an estimable between-group effect on pain. Taken together, we read the randomized evidence as dose-dependent rather than simply negative: both trials that used 6 × 1 Gy were neutral, whereas the only trial that tested 6 × 0.5 Gy against sham met its primary responder endpoint. Our position is that this is sufficient to justify offering LDRT at 3 Gy in six 0.5 Gy fractions now, outside a clinical trial, to patients aged ≥50 years with symptomatic OA refractory to conservative management who accept a small quantified late risk in exchange for a noninvasive intervention with no demonstrated organ toxicity, and to do so with prospective outcome capture. It is not sufficient to support a guideline-level recommendation for routine use, to support continued use of 6 × 1 Gy regimens, or to support treatment of patients younger than 40 years. Figure 4 presents an evidence and risk-benefit map of major OA interventions.

**Figure 3:**
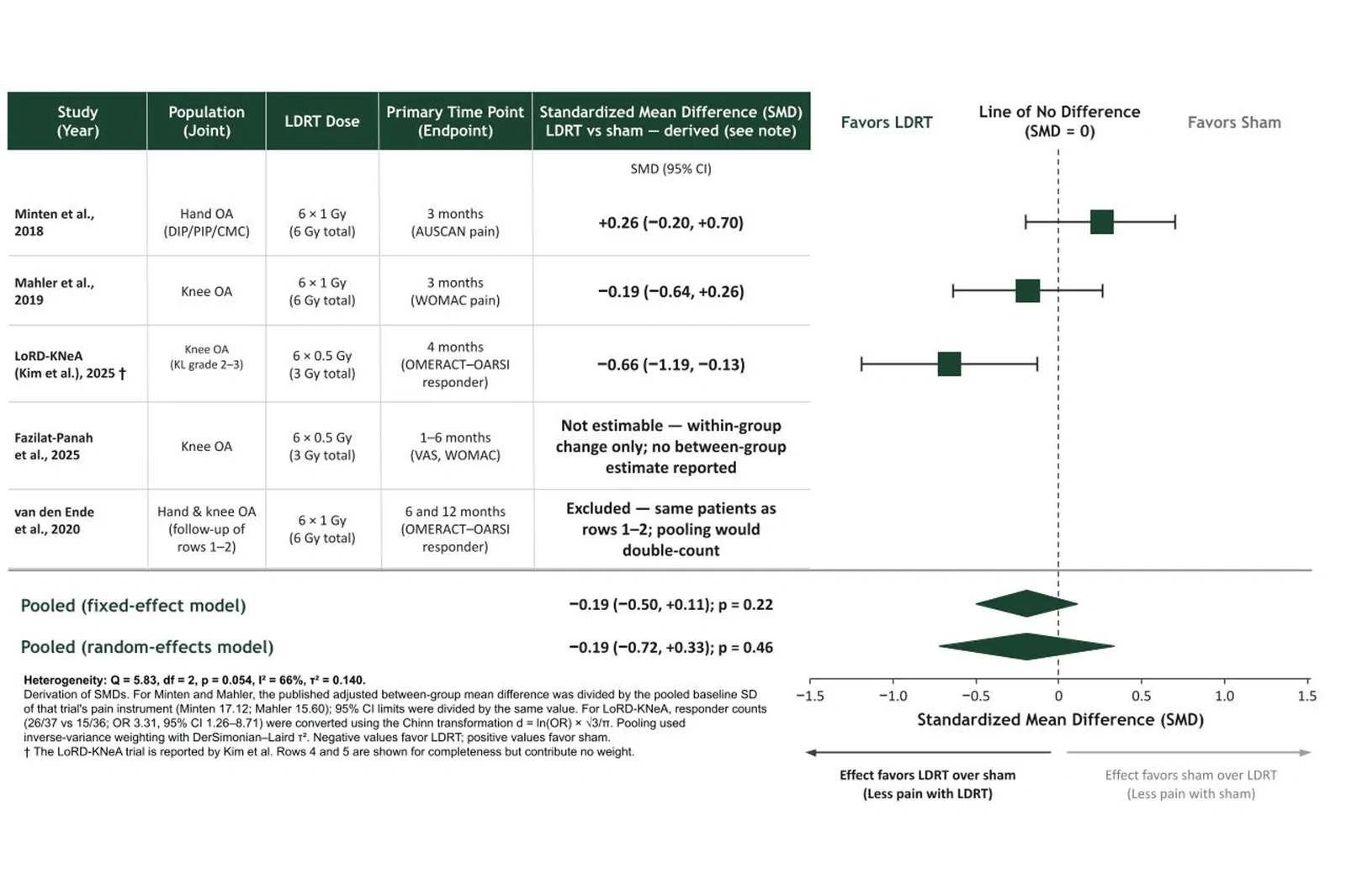
Forest plot of randomized sham-controlled trials of low-dose radiation therapy in osteoarthritis. Forest plot of the randomized sham-controlled trials of low-dose radiation therapy in hand or knee osteoarthritis that report an estimable between-group effect on pain. Squares represent the point estimate of the standardized mean difference and horizontal lines the 95% confidence interval, with square size proportional to study weight; diamonds represent the pooled estimate under fixed-effect and random-effects models, and the dashed vertical line marks the line of no effect. Negative values favor low-dose radiation therapy and positive values favor sham. Standardized mean differences were derived as detailed in the main text and in the note printed within the figure. Fazilat-Panah et al. report only within-group change and are not estimable; the van den Ende extended follow-up reports the same cohorts as rows 1 and 2 and is excluded from pooling to avoid double-counting patients. The pooled standardized mean difference was -0.15 (95% CI -0.42 to +0.12; p = 0.28) under the fixed-effect model and -0.18 (95% CI -0.69 to +0.32; p = 0.48) under the random-effects model, with Q = 6.78, df = 2, p = 0.034, I² = 71%, and τ² = 0.140. The pooled randomized estimate is therefore not statistically significant and is directionally inconsistent, dominated by the contrast between the two neutral 6 × 1 Gy trials and the positive 6 × 0.5 Gy LoRD-KNeA trial. †The LoRD-KNeA trial is reported by Kim et al. Abbreviations: CI = confidence interval; df = degrees of freedom; I² = between-study heterogeneity; LoRD-KNeA = low-dose radiation therapy for patients with knee osteoarthritis trial; Q = Cochran Q statistic; SMD = standardized mean difference; τ² = between-study variance. References: [56-57,61] Image credit: original figure created by the authors using Microsoft PowerPoint and Google Slides.

**Figure 4:**
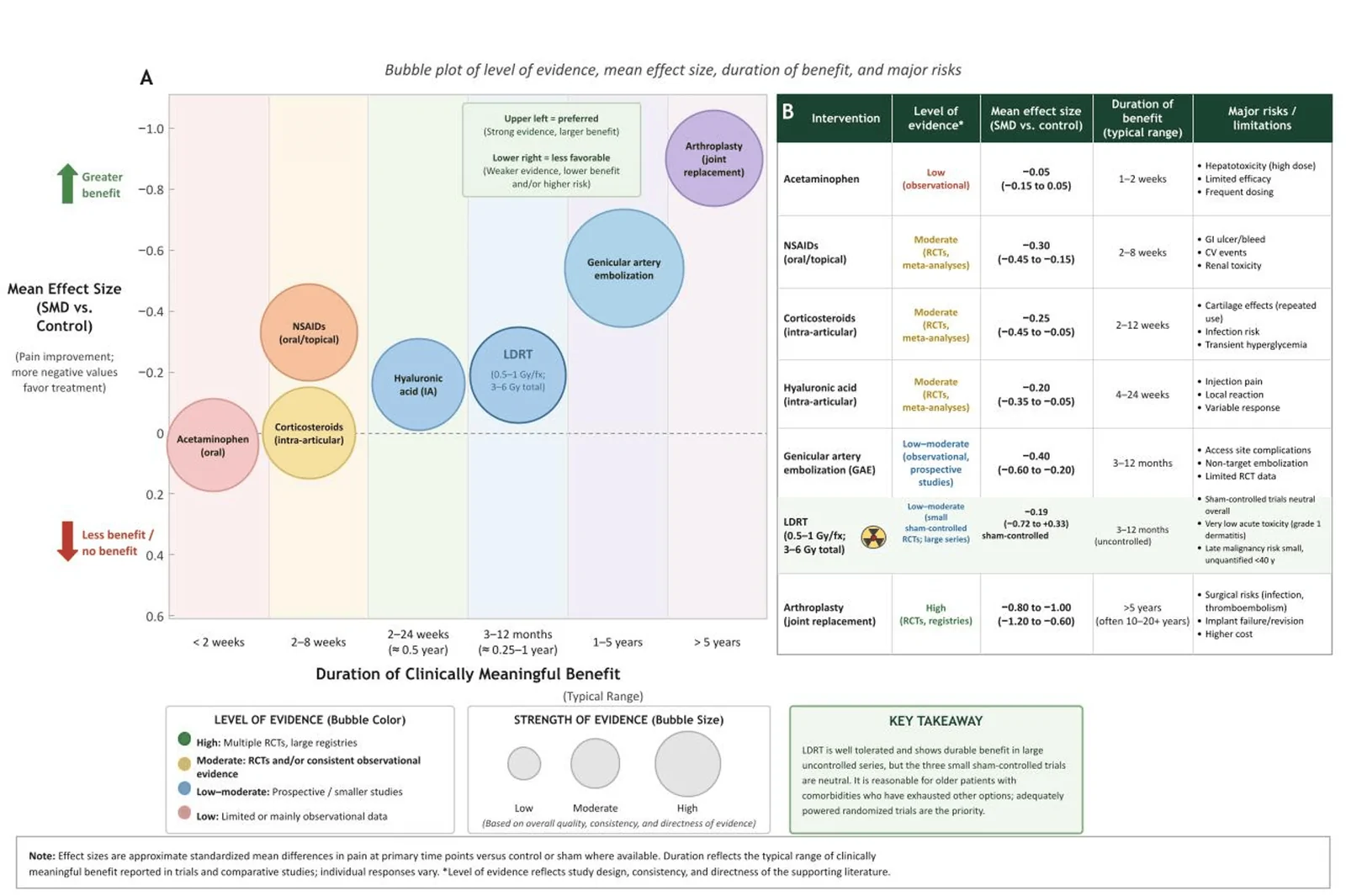
Evidence and risk-benefit map for major osteoarthritis interventions. Panel A is a bubble plot positioning seven osteoarthritis interventions by duration of clinically meaningful benefit (horizontal axis) and mean effect size for pain, expressed as the standardized mean difference versus control (vertical axis). More negative values favor treatment, so interventions in the upper left combine stronger evidence with larger benefit. Bubble color encodes level of evidence and bubble size the overall strength of evidence, as defined below the plot. Panel B tabulates the same interventions with level of evidence, mean effect size with 95% confidence interval, typical duration of benefit, and principal risks or limitations; the LDRT row is shaded. Effect sizes are approximate standardized mean differences in pain at primary study time points versus control or sham where available; individual responses vary. The low-dose radiation therapy effect size shown reflects uncontrolled and single-arm cohorts; the sham-controlled randomized estimate is smaller and non-significant (-0.19, 95% confidence interval -0.72 to +0.33) and is presented in Figure 3. The level of evidence for low-dose radiation therapy is accordingly moderate for symptomatic benefit in uncontrolled data but low to moderate for a sham-controlled effect, and the two should not be conflated. *Level of evidence reflects study design, consistency, and directness of the supporting literature. Abbreviations: CI = confidence interval; CV = cardiovascular; fx = fraction; GAE = genicular artery embolization; GI = gastrointestinal; Gy = gray; IA = intra-articular; LDRT = low-dose radiation therapy; NSAIDs = nonsteroidal anti-inflammatory drugs; OA = osteoarthritis; PRP = platelet-rich plasma; RCTs = randomized controlled trials; SMD = standardized mean difference. Image credit: original figure created by the authors using Microsoft PowerPoint and Google Slides.

Systematic Reviews and Meta-Analyses

A systematic review of radiotherapy for benign skeletal disorders concluded that LDRT appears highly effective in uncontrolled series, including OA, but noted the shortage and heterogeneity of randomized evidence [9,65]. The most OA-specific synthesis to date, Hammadeh et al.'s 2025 systematic review and meta-analysis, pooled RCTs and observational studies of LDRT in OA [66]. That review included 12 studies and 1,750 participants, of which 6 contributed to pooled analyses [66]. Across sham-controlled trials, LDRT did not significantly outperform sham for average pain at short or intermediate follow-up (pooled standardized mean difference -0.92; p=0.13) or for function (standardized mean difference 0.22; p=0.22), consistent with the neutral findings of Minten, Mahler, and van den Ende; adverse events were more frequent with LDRT than with sham (relative risk 1.44, 95% CI 1.08-1.92), driven largely by nail reactions in hand OA (relative risk 2.24, 95% CI 1.13-4.45), and the authors concluded that LDRT should remain investigational pending better randomized data [56-58,66]. The review highlighted large within-group improvements in uncontrolled cohorts and substantial heterogeneity in dose, fractionation, patient selection, and outcome measures, and called for larger, stratified RCTs using biologically optimized regimens [66]. Quantitatively, our own pooling of the three sham-controlled trials with estimable between-group pain effects (Figure 3) yields Q = 5.83 on 2 degrees of freedom (p = 0.054), I² = 66%, and τ² = 0.140, confirming substantial statistical heterogeneity. This heterogeneity is not merely statistical noise: it maps directly onto a dose contrast, with the two neutral trials using 1 Gy per fraction and the single positive trial using 0.5 Gy per fraction, and onto differences in joint site, baseline pain eligibility thresholds, and whether the primary endpoint was a responder proportion or a mean pain score. These limitations of dose heterogeneity and patient selection give pause when assessing reproducibility and motivate the dose-finding and stratified designs in current and planned trials [61-62].

Dose, Fractionation, and Technique for LDRT in OA

European consensus recommendations from the German Society of Radiation Oncology (DEGRO) endorse standardized LDRT regimens for benign degenerative joint disease [21-22,65]. Typical prescriptions include 0.5-1.0 Gy per fraction, six fractions delivered two to three times per week, and a total dose of 3-6 Gy [21-22]. Many contemporary centers prefer 0.5 Gy × 6 (total 3 Gy), based on preclinical data suggesting maximal anti-inflammatory effects in the 0.3-0.7 Gy per-fraction range [6,9,21]. The DEGRO S2e guideline on radiotherapy of benign disorders is explicit about retreatment: a second series may be delivered after an interval of 6-12 weeks (6-8 weeks for the small joints of the hand, 10-12 weeks for the elbow) in partial or non-responders and is successful in more than 80% of such patients, whereas a third series is markedly less effective; the cumulative lifetime skin dose at any single site should not exceed approximately 25 Gy [21-22]. The Makarova trial in early-stage knee OA used a total dose of 4.5 Gy in 10 fractions of 0.45 Gy, at the lower end of the per-fraction range, and repeat treatment at the same site after symptom recurrence is supported both by clinical experience and by explicit DEGRO guidance [21-23].

Planning is technically straightforward, using 2D or basic 3D conformal techniques with low-energy photons [19,21,53]. For peripheral joints, computed tomography (CT) simulation is often unnecessary; opposed or single fields encompassing the symptomatic joint with modest margins are generally sufficient [19,43]. Shielding is not limited to the fingernails. DEGRO guidance requires lead shielding of the nail beds when the small joints of the hand are treated, and explicit gonadal shielding whenever the knee or hip is irradiated, since scattered dose to the gonads is the dominant contributor to stochastic risk for these fields; recommended lead thicknesses are 0.15 mm at ≤50 kV, 0.8 mm at >50-100 kV, 2.0 mm at 120-200 kV, and ≥4.0 mm at >200-300 kV, and shielding must be wrapped in plastic foil because backscatter from bare lead can double or triple the surface dose [21-22]. Beyond the nail beds and gonads, no additional organ-specific shielding, such as thyroid or breast shielding, is specified for peripheral joint LDRT, because these structures lie well outside typical treatment fields [21-22]. Image guidance can improve reproducibility but is not mandatory. Patients should be counseled about the time course of response, which differs substantially from that of an intra-articular injection. A minority of patients improve during the treatment course itself, and in the largest series, 60.0% of treated sites had responded by the final fraction, but most of the benefit accrues after treatment ends, with a 65.6% response rate at approximately eight weeks and further improvement documented at three to six months [19-20]. Patients should therefore be told explicitly that they may not feel better when they leave the department, that the expected interval to meaningful relief is roughly 6-12 weeks, and that a transient increase in joint pain during or shortly after the course is possible but uncommon and self-limited [19-20].

Patient Selection and Eligibility for LDRT

Drawing on DEGRO guidance, observational data, and trial inclusion criteria, a pragmatic selection framework for LDRT in OA includes [21-23]:

Age: ≥50 years is preferred and is the threshold used consistently throughout this review, including in the Abstract, the Comparative role section, and the Conclusions. Between 40 and 50 years, LDRT may be offered with careful counseling regarding theoretical long-term risk and only after conservative options have been exhausted. Patients younger than 40 years should not be irradiated as a rule, consistent with DEGRO guidance and with the steep age-dependence of the excess relative risk of radiation-induced malignancy, which falls from approximately 15 for exposure at ages 0-9 and 5.7 at ages 10-19 to 1.3 at ages 20-39 and approximately 0 at ages ≥40 [21-22].

Disease confirmation and severity: clinically and radiographically confirmed OA (Kellgren-Lawrence 2-4, or 0-2 with clear symptoms and clinical criteria as in the Makarova trial) with symptom-imaging concordance at the joint considered for treatment [23,43].

Symptom profile: persistent moderate-to-severe pain and functional limitation despite optimized conservative measures. We define moderate-to-severe pain as ≥40 mm on a 100 mm visual analog scale, the threshold used by the European Medicines Agency guideline on the clinical investigation of medicinal products for osteoarthritis and adopted in contemporary interventional OA trials; for reference, the LoRD-KNeA trial required walking pain of 50-90 mm and sham-controlled genicular artery embolization trials required >40 mm [61,67]. This eligibility threshold should not be confused with the minimal clinically important difference, which is approximately 20 mm on a 100 mm visual analog scale for pain on movement, nor with the patient-acceptable symptom state of approximately 32 mm in knee OA [68]. The OMERACT-OARSI responder criterion used as the primary endpoint in several LDRT trials requires only at least mild baseline pain and is defined as a ≥50% relative and ≥20-point absolute improvement in pain or function, or a ≥20% relative and ≥10-point absolute improvement in at least two of pain, function, and patient global assessment [61,68].

Prior therapy: not every modality listed below needs to be exhausted before LDRT is considered, and requiring sequential failure of all of them is neither evidence-based nor patient-centered. We regard an adequate trial of non-pharmacologic management (weight loss, exercise, physical therapy) as a genuine prerequisite, since it has the most favorable benefit-to-harm profile of any OA intervention and is recommended unconditionally by guidelines [1,12]. A trial of topical NSAIDs, and of oral NSAIDs where cardiovascular, gastrointestinal, and renal risk permits, is also reasonable [1,12]. By contrast, acetaminophen, intra-articular corticosteroids, and hyaluronic acid are best regarded as optional rather than mandatory prerequisites: acetaminophen produces effects far below the minimal clinically important difference and has been deprioritized in contemporary guidelines [2,24-25]; repeated corticosteroid injections may not exceed saline at two years and may accelerate cartilage loss, so mandating them before a non-invasive alternative is difficult to justify [15-16]; and hyaluronic acid has a pooled effect below the minimal important difference with no controlled data beyond six months [26-27]. Requiring documented failure of an intervention that is itself of marginal benefit, or that may cause structural harm, is not a defensible gate to a lower-risk option.

Surgical status: not scheduled for imminent arthroplasty; either poor surgical candidates or patients seeking to defer surgery.

Relative contraindications include age <40 years, pregnancy, active infection in or near the joint, prior overlapping high-dose radiation, and the anticipated need for repeated multi-joint courses in younger patients, where cumulative lifetime cancer risk becomes more important [21-22,69-70].

Comparative role of LDRT within OA management

When placed alongside standard OA therapies, LDRT is best understood as a non-pharmacologic, non-surgical option for patients with refractory symptoms who are not yet ready for, or are not ideal candidates for, joint replacement [1,10,19]. The case for offering it rests on a direct comparison of benefit, durability, and harm rather than on novelty. Acetaminophen yields a mean difference of roughly 3 points on a 0-100 pain scale against a 9-point minimal clinically important difference [2,24]. Hyaluronic acid yields a standardized mean difference of -0.08, below the minimal important difference, with no controlled data beyond six months and a higher rate of serious adverse events than placebo [26-27]. Corticosteroid injections give 1-6 weeks of relief, do not exceed saline at two years, and are associated with accelerated cartilage loss [15-16]. Oral NSAIDs provide approximately 10 mm of relief on a 100 mm scale for 2-4 weeks and raise myocardial infarction risk within the first week of use [29,37]. Genicular artery embolization did not differ from sham at either 4 or 12 months in the best available randomized data and carries procedural adverse events [45-46]. Against that comparison set, a single six-fraction course of LDRT delivering 3 Gy produced a 70.3% responder rate versus 41.7% for sham at four months in a randomized trial, has produced 60-80% response rates with benefit lasting months to years in large cohorts, has no organ-specific systemic toxicity, has no reported structural harm to cartilage, and carries an in-field second-malignancy rate of 0.064% in the largest long-term series [19-20,61,71]. The relevant question is therefore not whether LDRT is definitively proven, but whether demanding further proof of a non-invasive intervention while continuing to use interventions of demonstrably marginal benefit is internally consistent. Uncontrolled and prospective LDRT cohorts report pain response rates of 60-80%, with many patients experiencing benefits lasting months to a year or longer, and some improving further after repeat courses [19-20,55]. By comparison, acetaminophen produces small average analgesic gains [24], NSAID effects are modest and time-limited [29,33], corticosteroid injections typically deliver 1-6 weeks of relief [15-16], and HA effects are small and inconsistent [26]. On short-term placebo-controlled effect size, however, LDRT does not exceed oral or topical NSAIDs or intra-articular corticosteroids (Figure 1); its case rests on the reported durability of benefit and on its safety profile rather than on the magnitude of short-term analgesia.

Early sham-controlled RCTs of LDRT for OA did not demonstrate superiority over sham for hand or knee OA, but they were small, used a single 1 Gy × 6 regimen, and did not stratify by inflammatory phenotype [56-58]. More recent sham-controlled and pragmatic studies, along with the Makarova 9-year RCT and the LoRD-KNeA trial, indicate that under specific dosing and selection conditions, LDRT can provide benefits beyond placebo and may influence long-term structural and disability outcomes [23,60-61,63]. From a safety perspective, short courses of LDRT lack the gastrointestinal, renal, and cardiovascular toxicity associated with chronic systemic NSAIDs and avoid the infection and cartilage-damage risks of repeated intra-articular steroids [16,21-22,37-40]. Acute adverse effects are usually mild; when prospectively graded, the only reported events have been 11 grade 1 dermatitis events in a 108-patient, 150-joint prospective United States series, and nail reactions occur more frequently after hand LDRT than after sham [9,19,21-22,43,66,72]. Decades of European experience have identified only a very small number of in-field malignancies plausibly attributable to typical OA LDRT regimens, principally in older adults [9,19,21-22,43,71]. For balance, several U.S. payers still classify LDRT for OA as investigational; the randomized evidence is heterogeneous and dose-dependent, and the absolute number of arthroplasty events in the Makarova trial was small, so structural endpoints should be interpreted cautiously pending replication. We would nonetheless distinguish coverage status from evidentiary status. LDRT is not an unproven experimental technique: it has more than eight decades of clinical use, a defined and reproducible mechanism, at least one positive sham-controlled randomized trial at the modern dose, and a quantified late-risk profile. In our view, it is more accurately characterized as an established but under-evaluated therapy whose optimal dose and target population are still being defined, and it is reasonable to offer it in routine practice to appropriately selected older patients with prospective outcome capture.

Within a multidisciplinary framework spanning primary care, rheumatology, orthopedics, pain medicine, and radiation oncology, LDRT can be integrated as a consideration after failure or intolerance of standard non-surgical options and prior to, or in conjunction with deferral of, arthroplasty [23,30,60]. Figure 5 depicts a multidisciplinary clinical pathway integrating LDRT into OA management. Clear referral criteria, standardized outcome measures (e.g., WOMAC, KOOS, Patient-Reported Outcomes Measurement Information System (PROMIS) Pain), and participation in prospective registries or trials are essential to refine patient selection and strengthen the evidence base [73-76].

**Figure 5:**
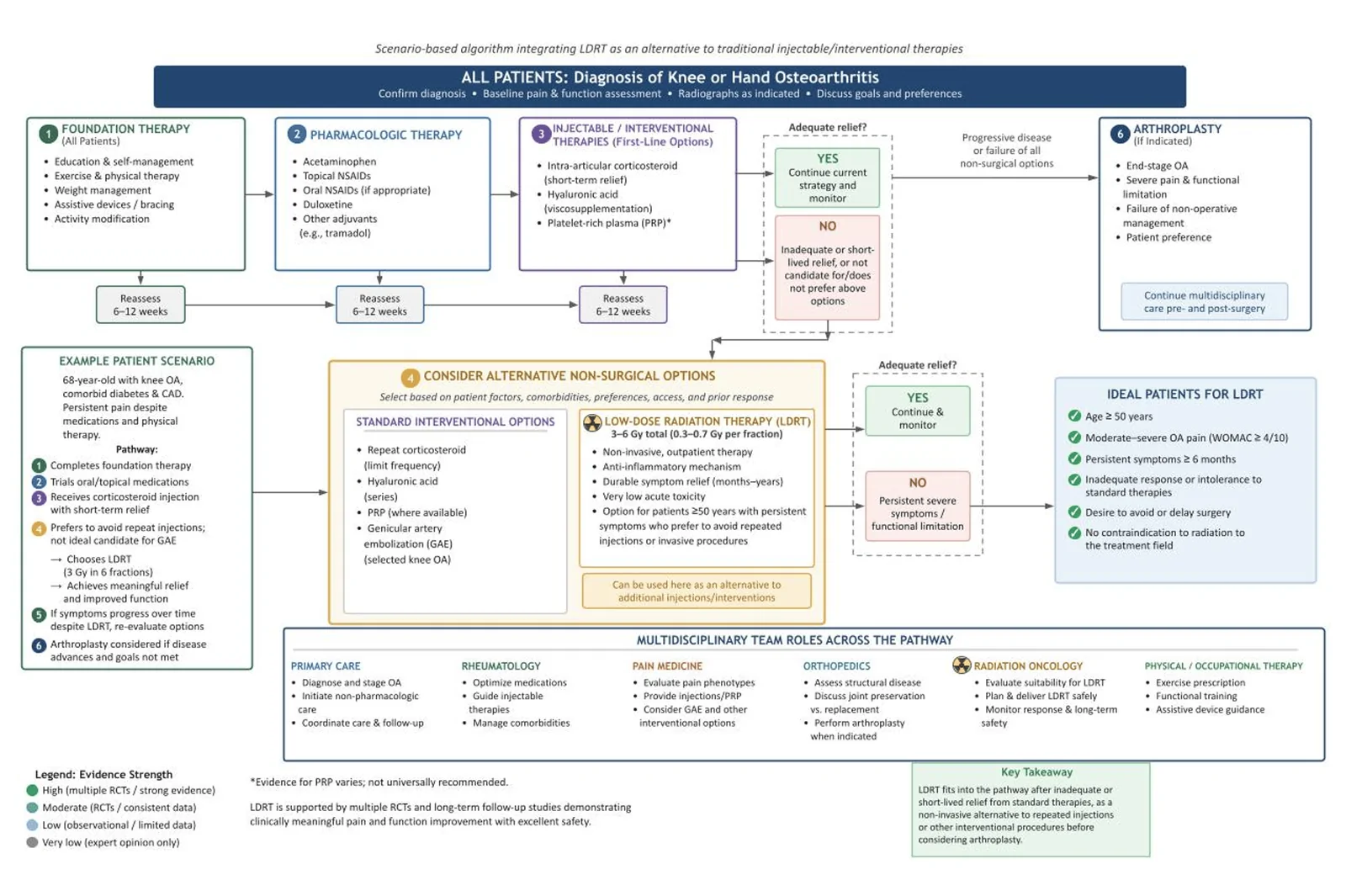
Multidisciplinary clinical pathway integrating low-dose radiation therapy into osteoarthritis management. Scenario-based clinical algorithm integrating low-dose radiation therapy as an alternative to traditional injectable and interventional therapies for knee osteoarthritis. The upper row shows the sequential care pathway: core non-pharmacologic and pharmacologic management for all patients, followed by injectable or interventional first-line options, reassessment at 6-12 weeks, and referral for arthroplasty if indicated. The middle section expands the decision point at which low-dose radiation therapy is considered, comparing other interventional options, including genicular artery embolization as an investigational option, with a low-dose radiation therapy course of 3-6 Gy total delivered in 0.3-0.7 Gy fractions, and lists the eligibility criteria favoring its use: age ≥50 years, moderate-to-severe pain of at least 40 mm on a 100 mm visual analog scale, radiographically confirmed knee or hand osteoarthritis, inadequate response or intolerance to standard therapy, a wish to avoid or defer surgery, and no contraindication to irradiation of the treatment field. The illustrative case narrative on the left follows one representative patient through the pathway to a course of 3 Gy delivered in six fractions. The lower band summarizes multidisciplinary team roles, abbreviations, and the key takeaway. *Evidence for platelet-rich plasma varies, and it is not universally recommended. Abbreviations: CAD = coronary artery disease; GAE = genicular artery embolization; Gy = gray; LDRT = low-dose radiation therapy; NSAIDs = nonsteroidal anti-inflammatory drugs; OA = osteoarthritis; PRP = platelet-rich plasma; RCT = randomized controlled trial; WOMAC = Western Ontario and McMaster Universities Osteoarthritis Index. Image credit: original figure created by the authors using Microsoft PowerPoint and Google Slides.

Safety, secondary malignancy risk, and guideline perspectives

Acute and subacute toxicity from OA LDRT is minimal; across large OA cohorts and benign skeletal series, serious acute adverse events are rare and generally limited to mild skin erythema or transient symptom flare [9,19,43]. Unlike systemic NSAIDs, LDRT does not confer direct gastrointestinal, renal, or cardiovascular risks, and unlike repeated corticosteroid injections, it does not increase infection risk or directly damage articular cartilage [16,37-40].

A major concern limiting broader LDRT use is the risk of radiation-induced malignancy. DEGRO guidelines on radiotherapy of non-malignant disorders note that typical OA regimens (total 3-6 Gy in small fields) confer an extremely small additional lifetime malignancy risk in older adults and that attributable second cancers have been exceedingly rare across four decades of clinical use in high-volume German benign-radiotherapy practice [21-22,77]. That statement now requires quantification rather than categorical denial. A long-term retrospective analysis of 4,699 patients treated with 6 × 1.0 Gy for non-malignant musculoskeletal disorders at a single German center between 1994 and 2010, first presented at the 2025 American Society for Radiation Oncology annual meeting and subsequently published in full, identified three in-field solid malignancies: one lung cancer 12 years after thoracic spine irradiation and two basal cell carcinomas at 8 and 13 years after shoulder irradiation, an absolute in-field incidence of 0.064% [71]. Hematologic malignancy-free survival was also lower in irradiated patients than in the comparison population at 12.5 years (99.0% versus 97.7%; p=0.002), although the absolute difference was small and confounding by indication cannot be excluded [71]. We therefore state explicitly that in-field second malignancies after LDRT for benign disease are not zero. They are rare, were of low lethality in the reported cases, and occurred after latencies of 8-13 years, which is directly relevant to counseling patients in their fifth and sixth decades and reinforces the guidance against irradiating patients younger than 40 years [71]. Risk modeling using International Commission on Radiological Protection (ICRP) frameworks suggests that for patients aged 50-60 years, the absolute excess lifetime cancer risk from a 3-6 Gy OA regimen is on the order of, or below, that of many accepted medical exposures and likely outweighed by potential gains in pain and function [21-22,69-70]. DEGRO and European consensus statements therefore recommend using LDRT predominantly in patients aged ≥40-50 years, avoiding very young adults, and individualizing decisions in middle-aged patients, particularly when multiple joints may require treatment [21-22].

In contrast, several North American payer policies classify LDRT for OA as investigational, citing negative early RCTs and calling for additional high-quality randomized data, while acknowledging that retrospective series show substantial pain improvement [78-79]. As contemporary RCTs, long-term cohort data such as the Makarova trial, and mechanistic studies accumulate, reassessment of these positions is warranted; in particular, the LoRD-KNeA result is the first sham-controlled randomized evidence at the 0.5 Gy per-fraction dose and postdates the negative early trials on which most existing coverage policies rest [23,61,78-79].

Multidisciplinary collaboration and clinical pathways

Successful integration of LDRT into OA care requires structured collaboration across specialties such as shared referral criteria, joint clinics, standardized outcomes, and patient education. 

Shared Referral Criteria

Rheumatology, orthopedics, pain medicine, and radiation oncology should agree on criteria for LDRT referral, including age, failure or intolerance of standard non-surgical therapies, and absence of an immediate surgical indication.

Joint Clinics and Conferences

Multidisciplinary OA boards or combined rheumatology-radiation oncology clinics can facilitate case selection, particularly for borderline patients or those with complex comorbidities.

Standardized Outcomes

Use of validated instruments (WOMAC [73], KOOS [74], the Functional Index for Hand Osteoarthritis [75], and PROMIS pain interference [76]) at baseline and prespecified time points (3, 6, and 12 months) enables consistent assessment and pooling of outcomes in registries and trials. These instruments should be described accurately: WOMAC comprises pain, stiffness, and physical function subscales and contains no quality-of-life subscale, whereas KOOS adds a four-item knee-related quality-of-life subscale; neither is a generic health-related quality-of-life measure [73-74]. Studies that report quality of life on the basis of WOMAC alone are in fact reporting function, and trials of LDRT in OA should pair a disease-specific instrument with a generic preference-based measure such as the EuroQol five-dimension questionnaire if quality-of-life or cost-utility claims are to be made [73-74]. We also note, for accuracy, that no formally labelled phase IV trial of LDRT in OA exists; the currently registered studies are phase II-III or observational and include IMMO-LDRT02 (NCT05887284, target enrollment 132, recruiting, estimated completion December 2028), Radio-KO (NCT06887829), LORAFA (NCT05852808) in facet-joint disease, the KORT protocol, and the prospective observational MOBILE cohort in the United States [61-62,72,80].

Patient Education

Rheumatologists and orthopedists should have access to decision aids describing LDRT's rationale, evidence, and risks, supporting shared decision-making [25,81-83].

Research priorities

To definitively establish or refute a role for LDRT in OA and clarify its optimal use, several research priorities emerge.

Firstly, large, adequately powered RCTs, where pragmatic multicenter randomized trials comparing standardized LDRT regimens (e.g., 0.5 Gy × 6) with sham in knee and hand OA, powered to detect small-to-moderate effect sizes, with 12-24-month follow-up and prespecified responder analyses [60-62].

Secondly, phenotype-driven stratification, wherein trials should stratify by inflammatory phenotype (e.g., synovitis on ultrasound or MRI, C-reactive protein), given mechanistic and clinical signals that LDRT may be more effective in patients with active inflammation.

Thirdly, dose-finding studies involving randomized comparisons of 0.3, 0.5, and 0.7 Gy per fraction can define the optimal balance between efficacy and theoretical risk, informed by LoRD-KNeA dose-stratified data [61].

Fourthly, biomarkers and mechanistic endpoints that integrate blood and synovial biomarkers, immune profiling, and advanced imaging into clinical trials to develop predictive and pharmacodynamic markers of response.

Additionally, no formal cost-effectiveness or cost-utility model of LDRT in OA has been published. Analyses comparing LDRT with repeated injections, chronic NSAID or opioid use, and deferred arthroplasty in older adults are needed, incorporating quality-adjusted life years derived from a generic preference-based instrument, current reimbursement for each comparator, and the value of avoided or deferred arthroplasty [42].

Furthermore, long-term structural and arthroplasty endpoints require replication and extension of the Makarova trial in larger, multinational RCTs with knee or hip arthroplasty and radiographic or MRI structural progression as prespecified primary endpoints [23].

Finally, prospective registries tracking late events, including second malignancies, in patients treated with LDRT for OA, with particular attention to younger individuals and those receiving multiple joint courses [21-22,69-70].

## Conclusions

We conclude that LDRT, prescribed as 3 Gy in six 0.5 Gy fractions, should be offered as a legitimate non-surgical option to patients aged ≥50 years with symptomatic hand or knee OA who have failed non-pharmacologic management and, where tolerated, NSAIDs, who are not candidates for or wish to defer arthroplasty, and who accept a small, quantified, latency-dependent late risk. Treatment should occur within a multidisciplinary framework with prospective outcome capture using standardized instruments, and preferably within a registry or clinical trial. We stop short of recommending LDRT as routine standard care, of endorsing 6 × 1 Gy regimens, or of extending it to patients younger than 40 years, and we do not regard the arthroplasty- and disability-sparing signals as established. Concurrently, well-designed, adequately powered RCTs and translational studies, co-led by rheumatology, orthopedics, and radiation oncology, are urgently needed to confirm or refute long-term benefits such as reduced arthroplasty and disability and to inform updated guidelines.
