# Correction: Low-Dose Radiation Therapy for Osteoarthritis: A Critical Review and Proposal for Integration Into Contemporary Care

**DOI:** 10.7759/cureus.c497

**Published:** 2026-10-30

**Authors:** Kishan M Patel, Trevor Wilson, Jessica Schuster, Bobby Koneru, Tarita O Thomas

**Affiliations:** 1 Radiation Oncology, Northwestern University Feinberg School of Medicine, Chicago, USA; 2 Radiation Medicine, University of Wisconsin–Madison, Madison, USA; 3 Radiation Oncology, University of Wisconsin–Madison, Madison, USA; 4 Radiation Oncology, Stritch School of Medicine, Cardinal Bernadin Cancer Center, Loyola University, Maywood, USA; 5 Radiation Oncology, UW Health in Northern Illinois, Rockford, USA

Line 2 of paragraph 18 in Results has been changed to reflect a radiation dose of 0.05 Gy × 6 fractions (total 0.3 Gy), or 0.5 Gy × 6 fractions (total 3 Gy). Previously this was misstated as 0.3 Gy × 6 fractions (total 1.8 Gy), or 0.5 Gy × 6 fractions (total 3 Gy).

